# Identifying and assessing BIM implementation challenges and success factors in sustainable building projects among Malaysian SMEs

**DOI:** 10.1038/s41598-026-39021-5

**Published:** 2026-02-15

**Authors:** Yasser Yahya Al-Ashmori, Idris Othman, Al-Hussein M. H. Al-Aidrous, Ali Alashwal, Mohamud Ibrahim, Ahmed Farouk Kineber

**Affiliations:** 1 School of Engineering, Design and Built Environment, Western Sydney University, Penrith, NSW 2751 Australia; 2 Department of Civil Engineering, Universiti Teknologi PETRONAS, Seri Iskandar, 32610 Perak Darul Ridzuan Malaysia; 3 Hadramout Research Center, Al-Ahgaff University, Al-Mukalla, Yemen; 4 Department of Civil Engineering, College of Engineering and Petroleum, Hadhramout University, Al-Mukalla, Yemen; 5 School of Engineering, Design & Built Environment, Western Sydney University, Penrith, NSW 2751 Australia; 6 Department of Infrastructure Engineering, University of Melbourne, Melbourne, VIC Australia; 7 College of Project Management, Built Environment, Asset & Maintenance Management (CoPBA), School of Engineering and Technology, Central Queensland University, Melbourne, VIC, Australia

**Keywords:** Building information modelling, Sustainability, BIM challenges, BIM critical success factors, BIM implementation, Empirical study, Civil engineering, Sustainability

## Abstract

Building Information Modeling (BIM) is considered a crucial technological innovation that should be embraced. While BIM adoption has progressed among large firms, its implementation within Small Medium Enterprises (SMEs) remains limited, particularly in developing countries where SMEs dominate the construction sector. Construction SMEs face unique constraints, including limited financial resources, fragmented project teams, restricted technical capacity, and lower organizational readiness, making BIM adoption both critical and challenging. The aim of this research is to investigate the challenges and key success factors for BIM implementation from Malaysian SMEs. A survey was distributed to 590 construction firms, resulting in 268 usable responses. The results showed that only 13% of respondents reported using BIM in their building projects, with the majority (76%) citing complicated legal framework, lack of standardized BIM processes, absence of affordable training, low levels of trust and fragmented team. For effective Implementation of BIM, the top critical success factors included early involvement and participation of project teams, availability of information and technology, and early selection of appropriate BIM tools. The outcomes provide targeted insights for SMEs and policymakers by offering empirically validated guidance to support BIM adoption in sustainable building projects. While the study is based on Malaysian context, the results are relevant to other developing nations with SME-dominated industry facing similar institutional and technological conditions. The findings provide targeted insights for SMEs, policymakers, and industry stakeholders by offering empirically validated guidance to support BIM adoption in sustainable building projects.

**Supplementary Information:**

The online version contains supplementary material available at 10.1038/s41598-026-39021-5.

## Introduction

The building sector significantly influences public health and well-being, while also serving as a fundamental contributor to a nation’s socio-economic development^1^. In developing nations, the building industry has witnessed substantial transformations aimed at fulfilling local economic objectives^2,3^. In these countries, building endeavours commonly encounter a multitude of obstacles such as incomplete execution, delays in schedules, exceeding budgets, inadequate quality, and a substantial risk of falling short of intended objectives^4,5^. The remarkable progress witnessed in these nations underscores the necessity of residential structures that fulfil fundamental living requirements^6^. As a result, governments have prioritized the implementation of multiple affordable housing regulations to ensure the availability of housing that is affordable for all^1^. Estimations indicate that over 250 large-scale construction projects are underway in developing nations, scheduled for completion by 2030^7^. Despite this growth, the sector continues to struggle with aligning its practices to international sustainability benchmarks, thereby limiting its potential for enhanced efficiency and global competitiveness.

Autodesk^8^ argued that BIM is an advanced, model-based 3D methodology that equips engineers across the Architecture, Engineering, Construction, and Operation (AECO) sectors with essential insights and tools enhancing the planning, design, construction, and management of the building industry. BIM inherently improves building design, construction, and maintenance processes^9–11^. In response to the demands of stakeholders in the field, Building Information Modelling (BIM) undergoes continuous and significant transformation to address the persistent challenges of productivity, time efficiency and cost management^12^. It also facilitate the interaction within management, information, and processes^13^. Moreover, advancements in Building Information Modelling (BIM) have led to the adoption of numerous tools aimed at enhancing the overall success of building projects^14^. Consequently, BIM has been recognized as a vital technology for lifecycle management, offering substantial potential to influence every phase of a building project^15,16^.

While there are numerous advantages associated with this approach, it is conceivable that the full potential of BIM may not yet be fully realized^17^. Hence, there is a lack of detailed obstacles when a company starts to adopt BIM within the building industry of developing countries^18^. Although prior studies have extensively examined the technical effectiveness and application of BIM in many developed nations, limited effort has been dedicated to investigate the challenges associated to BIM implementation^17^ and how to consolidate BIM enablers to support BIM implementation within developing nations.

Moreover, there has been a lack of significant academic focus on overcoming these challenges to BIM, despite the crucial importance of comprehending BIM implementation challenges in order to enhance its usage in the construction sector^17^. Thus, the aim of this is to explore the obstacles and Critical Success Factors (CSFs) related to BIM adoption. The specific goals include identifying and prioritizing BIM challenges to establish the necessary conditions for the effective application in building projects across developing countries. By doing so, this research support industry stakeholders in enhancing project success, minimizing inefficiencies, and improving overall quality through BIM implementation. This study is expected to provide significant benefits to various industry professionals, including project bidders, policy makers, and architects^19^. In the context of Malaysia and other developing nations engaged in similar construction projects, this research aims to contribute meaningful recommendations for improving BIM adoption^20^. Additionally, this study presents empirical findings that provide crucial elements for achieving success, guiding the industry, and overcoming prevailing challenges.

In Malaysia, the construction industry is predominantly composed of Small and Medium Enterprises (SMEs), which are responsible for delivering the majority of mid- and low-rise building projects. SMEs are formally classified based on a combination of paid-up capital and workforce size. According to national guidelines and industry practice, construction SMEs are defined as firms with paid-up capital not exceeding RM750,000 ($184,433) and employing less than 30 as small and not exceeding 75 employees as medium enterprise^21,22^.

Despite the dominant role of construction SMEs, they often operate under significant constraints, including limited financial capacity, reduced access to advanced digital technologies and fragmented organizational structures^23^. These constraints directly influence their ability to adopt and implement Building Information Modelling (BIM) effectively. Unlike large construction firms, SMEs typically lack dedicated BIM teams, in-house digital expertise, and the financial resilience required to absorb the high initial costs associated with BIM software, training, and process re-engineering. As a result, BIM adoption among SMEs remains slow, uneven, and vulnerable to falling behind national digitalization agendas and sustainability targets. Recent studies on construction SMEs have largely focused on developed countries, such as New Zealand and England, where mature regulatory frameworks and institutional support influence implementation outcomes^24,25^. Other research has relied on systematic literature reviews to conceptualize BIM adoption in SMEs, offering limited empirical validation at the organizational level^26^. Yet, a published paper conducted in Nigeria, have used a questionnaire to identify the barriers of BIM adoption among SMEs but have not examined the critical success factors required to overcome these challenges^27^. As a result, findings from these studies cannot be directly transferred to construction SMEs operating in resource-constrained environments. Thus, this study examines both BIM implementation challenges and critical success factors from the perspective of construction SMEs in Malaysia, thereby extending existing BIM literature with balanced and context-specific insights.

Additionally, the importance of overcoming BIM challenges and obtaining success factors is crucial. The majority of earlier BIM-challenges and CSF research focused on construction companies in general, high-rise projects, or in government-driven construction^10,28,29^. Even though SMEs constitute more than half of the industry players and are in charge of most mid- and low-rise building projects, prior studies seldomly ever focused on the SME segment. This study focuses on SMEs working in mid-rise and low-rise building projects as under-studied sector. Moreover, there are no previous efforts have evaluated the challenges and success factors simultaneously for SMEs adopting BIM^30^. Thus, to bridge this gap, this study aims to provide empirical evidence on the success factors which can mitigate the primary challenges for SMEs adopting BIM. Additionally, the empirically validated and reduced lists of BIM challenges and success factors into three challenge components and five success factors components are directly useable by SMEs and policy makers. Furthermore, this research findings provide practical recommendation to guide industry practitioners in optimizing BIM implementation while overcoming existing challenges. This study also provides empirical results that offer the elements for success to guide the industry and overcome existing challenges.

## Research background

In developing countries, the construction industry is undergoing into gradual digital transformation and Building Information Modelling (BIM) has become a cornerstone technology for achieving integration across design, construction, and facility management phases^31^. While BIM adoption has advanced rapidly in developed economies, its diffusion within developing countries has remained slow and uneven, particularly among small and medium-sized enterprises (SMEs) that dominate the sector^24^.

In the Malaysian context, the government has actively promoted BIM adoption through national strategies and the Construction Industry Transformation Programme^32,33^. Nevertheless, empirical evidence indicates that implementation remains concentrated within large or high-profile projects, leaving SMEs and mid- to low-rise building developments lagging behind^30^. This disparity threatens to deepen the technological divide within the industry, reducing the competitiveness of smaller firms and hindering national digitalization goals.

Existing literature has discussed the barriers and critical success factors (CSFs) influencing BIM adoption; however, most studies are either contextually generic without addressing the specific operational constraints of SMEs; or conceptually repetitive reconfirming well-known challenges (e.g., cost, training, interoperability) without producing empirically validated groupings that explain how these barriers interact and explain each other^32,34,35^. Furthermore, the SMEs segment, which forms the bulk of urban development in developing countries, has received limited scholarly attention. That includes projects typically involve smaller budgets, less sophisticated design teams, and decentralized decision-making structures—conditions that fundamentally alter how BIM is perceived and applied. Hence, there remains a significant gap in understanding BIM implementation within SMEs and the interplay between BIM challenges and success factors within this crucial segment of the industry.

Malaysia represents a relevant and important context for examining BIM implementation among construction SMEs. Although national initiatives and policies have promoted BIM adoption, implementation has been largely concentrated in large-scale and government-led projects, with limited penetration among SMEs. Construction SMEs differ fundamentally from large firms in term of risk tolerance, company scale and technological resources. Thus, BIM implementation challenges and success factors identified in large firms cannot be transferred directly to SMEs without empirical validation. This creates a critical research gap, as findings derived from large firms or mega-projects may not be directly applicable to SMEs operating in mid- and low-rise building projects. Therefore, an SME-focused investigation is necessary to generate context-specific empirical evidence that reflects the operational realities of this dominant yet under-researched segment of the construction industry.

### Challenges of BIM implementation

Aranda-Mena et al.^36^ identify software compatibility as a key obstacle to the adoption of BIM. Ku and Taiebat^37^ further highlight the lack of seamless interaction between different BIM software, necessitating the manual transfer of data between platforms rather than enabling direct interoperability^17^. The fundamental objective of using BIM is to achieve interoperability, which ensures smooth and efficient communication between various systems. This interoperability is essential for fostering collaboration among stakeholders throughout the entire lifecycle of a building project^38^. Despite its potential, existing research indicates that BIM software provides limited support for Small and Medium Enterprises (SMEs)^24^. Additionally, the unique data structures of BIM models have raised legal concerns, particularly regarding ownership rights over the extensive design, manufacturing, analysis, and construction information contained within them^39^. Another critical challenge in BIM implementation pertains to assigning responsibility for design accuracy. Unlike conventional design procedure, where professionals like architects, engineers, and others bears clearly defined responsibilities for their respective contribution, yet the integrated and collaborative nature of BIM, complicates the determination of liability for design errors^36^. However, in the case of BIM, determining such accountability is not as straightforward^16^.

Prior research, including Chan^29^, have highlighted the shortage of qualified professionals as a major barrier to the successful implementation of BIM. According to Aranda-Mena, et al.^36^ argue that in regions where skilled personnel are lacking, discussions about the benefits of BIM become irrelevant, as there are no professionals available to execute its implementation. Hence, in areas with a deficiency of trained experts, achieving effective BIM adoption remains an unattainable goal. Nonetheless, Sebastian^40^ expressed the belief that utilizing BIM for projects becomes challenging by poorly coordinated contract procedures, as the overall contractual process lacks proper incorporation of modern technology^17^. Furthermore, addressing these personnel and contractual challenges is essential for fostering a collaborative project environment leading to successful BIM implementation. If contractual processes are not clearly defined, BIM cannot be seamlessly incorporated If the procedures are not adequately defined, as it necessitates incorporating BIM implementation into the contract right from the outset^41^. Numerous businesses have hesitated to adopt BIM due to the significant adjustments that need to be made before its successful implementation^42,43^. The primary modifications that businesses encounter when adopting the BIM concept during design phase revolve around the extensive utilization of a shared building model and integrated coordination of these models throughout building process. These changes underpin the redefinition of workflows and collaborative practices within the BIM environment^37^.

Despite existing challenges, many experts continue to view BIM as a good substitute to conventional construction methods, arguing that traditional processes remain effective in smaller projects and do not necessarily require replacement^37^. However, in many developing nations, such as in Nigeria, lacks dedicated government legislation aimed at promoting BIM adoption and fostering a deeper understanding of its benefits^17^. This regulatory gap contrasts sharply with the policies in industrialized countries including the US, China and the UK, where governmental support has played a crucial role in advancing BIM implementation^37,44^. The absence of a dedicated body overseeing BIM adoption has hindered efforts within private sectors, especially considering that the government is the owner of all public projects. Consequently, the government is expected to take the lead in order to encourage other stakeholders to follow suit^44^. In Saudi Arabia, BIM adoption is hindered by a lack of government-driven initiatives and limited research focus on the subject^45^. Likewise, Saudi Arabian construction companies encountered challenges such as minimal engagement of client and stakeholders, shortage of expertise within BIM groups, and the lack of structured mentorship program led by an experienced BIM champion^46^.

The complexity of adopting or utilizing BIM extend beyond technical implementation and include issues like design accountability, ownership claims, patent rights, responsibility for creating and managing BIM. Furthermore, the financial burden of adopting BIM, along with the challenge of cost allocation among project stakeholders, remain a significant concern^23^. Gamil and Rahman^47^ identified additional obstacles to BIM adoption include financial constraints, limited awareness and understanding of BIM methodologies, lack of recognition of its benefits, and insufficient government support. Overall, the successful BIM implementation is hindered by range of persistent challenges. These challenges are often context-specific and influenced by geographical setting, economic status of a nation, government policies, and resistance to change. Table 1 provides a condensed overview of these obstacles.

**Table 1:** BIM challenges in building projects.

ID	Item Adopted from Al-Ashmori et al.^30^ work	Supported references
CF1	Creating demand for BIM projects or prioritizing BIM projects as a marketing brand.	^28,34,35,48^
CF2	Utilization of current contracts to fulfill BIM projects requirements.	^32,34,35,49,50^
CF3	Development of protocols for BIM standard modeling.	^48^
CF4	Developing a securing property assurance of BIM project information.	^32,49,51,52^
CF5	Convincing organizations and individuals to openly share information.	^32,52^
CF6	Build trust towards BIM technologies and overcome resistance factors.	^28,32,34^
CF7	Development of execution procedure and legal frameworks for BIM implementation.	^32,34,35^
CF8	Creating affordable training programs.	^28,32,35,48–50,52–54^
CF9	Minimizing the initial costs associated with BIM implementation.	^31,48,49,52,54^
CF10	Enhancing level of understanding of BIM technology and process implementation.	^28,32,35,50,52^
CF11	Standardizing BIM process and defining guidelines for its implementation.	^35,48,52^
CF12	Provision of comparative analysis between traditional and BIM-based projects as evidence.	^48^
CF13	Overcoming the constraints of limited BIM software tools and compatibility issues.	^32,48,52^
CF15	Building trust among BIM project teams and bridging the gap of work fragmentally.	^32,48,52,53^
CF16	Enhancing the Individual and group motivation to use BIM.	^28,32,34,48,52,54,55^
CF17	Understand BIM model interoperability mechanism among different BIM software.	^28,32,35,48,52^
CF18	Creating a platform for a collaborative working environment.	^32,52^
CF20	Setting out an efficient mechanism for coordinating BIM models.	^32,35,49,52,56^
CF21	Enhancing communication process among different parties.	^28,32^
CF22	Boosting the decision-making process among stakeholders.	^32,52,53,56^

### Enablers (critical success factors) for effective BIM implementation in building projects

In construction sector, poor document and information management are prevalent, resulting in detrimental effects on the project lifecycle^57^. Saka and Chan^58^ highlighted the industry’s reluctance to adopt modern digital technologies like Building Information Modelling, which has hindered its growth and modernization. However, recent years have witnessed a significant increase in BIM adoption, establishing it as a pivotal tool for design and construction across the global built environment^59,60^. As a solution, BIM has evolved with considerable potential to create, integrate, and sustain interconnected databases that store essential facility information, thereby supporting operations and maintenance processes^61^. Furthermore, Nieto-Julián, et al.^62^ further emphasized BIM’s role in enhancing data interoperability, particularly within multidisciplinary teams in the heritage sector. Olanrewaju et al.^57^ study identified four key drivers for BIM adoption in Nigeria: (1) construction-related considerations; (2) process digitalization and economic factors; (3) sustainability and operational efficiency; and (4) improvements in visualization and productivity. Stransky and Dlask^63^ argued that serves as a valuable decision-support tool during project execution, contributing to increased productivity. Furthermore, numerous studies have acknowledged BIM’s contribution to fostering collaboration among project stakeholders, particularly in areas like cost estimation and financial management^9,64,65^. BIM has increasingly been acknowledged as a pivotal tool in promoting sustainable construction practices, commonly referred to as “Green-BIM.” By utilizing BIM technology, the environmental impacts of construction activities can be effectively minimized^66^. Moreover, BIM can facilitate in conducting Building Life Cycle Assessment (LCA), offering valuable insights and recommendations to optimize BIM-LCA applications^67^. Additionally, the inherent visualization capability of BIM serves as a significant factor driving its adoption, allowing clients to virtually visualize their intended structure prior to commencing construction^57^. Through this interactive visualization, design teams can incorporate client feedback and make informed modifications to building features as needed^9,64^.

Additionally, Lin and Hsu^68^ highlighted the application of BIM in problem identification and management by integrating it with a web-based API. This implementation allows project stakeholders to visualize and monitor issues, as well as track construction progress in the early stages. Table 2 presents a concise summary of key BIM enablers identified from the literature.

**Table 2:** BIM enablers in building projects.

ID	Item Adopted from Al-Ashmori et al.^30^ work	Supported references
CSF1	Existence of procedures, frameworks, and guidelines.	^28,32,50,52,54,69,70^
CSF2	Develop research to identify changes with BIM implementation.	^52,69^
CSF3	Linking current policy with the BIM implementation requirement.	^35,49,52,71–73^
CSF4	Define team roles and responsibilities.	^70,71^
CSF5	Create BIM business opportunities and market support.	^32,74^
CSF6	Readiness of government and organization to reward for self-development skill in BIM technology implementation.	^28,32,34,52,54,70–72,74^
CSF7	Ability to allocate sufficient financial resources to invest in BIM development.	^28,32,49,52,54,69–72,74^
CSF8	Top management support to implement BIM.	^32,34,54,69–74^
CSF9	Ability to accommodate changes and upgrade to BIM-based system.	^70,72,75^
CSF10	Compatibility of BIM systems to support interoperability and collaboration.	^32,52,69,72,74^
CSF11	Availability of BIM systems/ tools/ extensions to support BIM implementation.	^75^
CSF12	Availability of Securing intellectual property and cyber security of BIM outcomes.	^32,49,51,52^
CSF13	Insure continues development to fulfill technology participant expectations.	^72^
CSF14	Knowledge and experience level of “players” in the BIM process and what are their drivers.	^52–54,71,72,74,75^
CSF15	Collaboration and readiness to share knowledge, risks, and reward.	^28,32,35,49–53,69,70,72,76]^
CSF16	Clear understanding of client requirements when using BIM in the project.	^52,70–73^
CSF17	Early involvement and participation of project teams.	^52,53,70,72–74^
CSF18	Mutual trust, respect, and personal commitments to cooperation.	^28,32,34,70,72^
CSF19	Ability to define external stakeholders’ potential impact on projects.	^32,49,75^
CSF20	Ability to understand each stakeholder’s interests.	^32,49,50^
CSF21	Ability to define a suitable way to manage stakeholder needs and wants.	^35,54,72^
CSF22	Active communication systems with appropriate stakeholders.	^28,32,35,49,52,56,70,73^
CSF23	People’s knowledge and awareness of the BIM system and its application.	^34,50,51,69,71,73,74^
CSF24	Ability to differentiate between different BIM software systems.	^28,32,54,74^
CSF25	Capability to use a BIM software tool.	^75^
CSF26	Understanding the mechanism of BIM execution through the project life cycle.	^52–54,74^
CSF27	Ability to manage information in a structured manner in a 3D environment.	^52,69,71–73^
CSF28	Knowing the usage of the multidisciplinary models that promotes collaborative processes.	^32,71–73^
CSF29	Availability of information and technology.	^69,71^
CSF30	Early selection of adequate project delivery method.	^52,70,72^
CSF31	Early selection of the appropriate BIM tools to perform the task.	^52,71,72^
CSF32	Understanding BIM project scope and contract agreement.	^52,72^
CSF33	Design BIM coordination strategy among project parties.	^32,70–73^
CSF34	Develop an intelligent 3D model that can be used by other disciplines.	^70,72,73^
CSF35	Produce models with different levels of development LOD100-LOD500.	^70,72,73^
CSF36	Produce models that can generate auto shop drawings for construction and fabrication.	^70,72,73^
CSF37	Visualize layout for site management, supervision, safety management, and quality management.	^70,72^
CSF38	Produce accurate model-based documentation through the project lifecycle.	^75^
CSF39	To be able to identify risks associated with bidding BIM projects (types, size, teams, and locations).	^52,70–72^
CSF40	Availability of effective communication methods.	^28,32,35,49,52,56,70,73^
CSF41	BIM process re-engineering and decentralized decision-making.	^72^
CSF42	An early formulation for collaborative method between stakeholders.	^28,32,35,49,50,56,75^
CSF43	Availability of effective project monitoring processes.	^70,75^
CSF44	Identify and produce BIM deliverables at each phase of the project’s life cycle.	^54,72,75^
CSF45	Determine and employ innovative ideas for collaborative practices.	^32,52^

## Methodology and data analysis approach

A systematic methodology approach was adopted in this research which depends on critical analysis of the existing literature review on BIM technology. The methodology concept of the factors identified based on synthesizing the BIM adaptation and implementation in terms of risks, challenges, barriers, influence factors, and success factors^77^. A questionnaire survey was selected as the primary data collection method due to its suitability for capturing perceptions and experiences from a large and geographically dispersed population of construction SMEs. Survey-based approaches are widely adopted in BIM adoption research as they enable the systematic evaluation of multiple factors influencing implementation across different contexts^32,51,52^. Alternative qualitative methods such as interviews were considered and used to prepare the adopted list of factors. The refined list of challenges and success factors were adopted from a previous exploratory study by the authors^30^ and used as the starting point. The list was refined via a targeted literature cross-check and validated by a focused expert meeting with three industry professionals who judged item relevance and wording prior to questionnaire finalization. Thus, this refined list of challenges and success factors are adopted. Based on critical literature analysis and experts’ opinions, the authors have identified the factors. Therefore, the authors have used those factors to perform a factor analysis study and factor naming based on survey questionnaire distributed to local building organization which categorized as small and medium enterprises (SMEs). The participated organizations are from mid and low-rise building sector. In this study, a questionnaire survey is used to empirically investigate the challenges and success factors of BIM implementation in construction SMEs in Malaysia. This method was adopted because it is being recognized as one of the most effective approaches to possibly reach a large population of respondents with relative ease^78^. It has been also adopted by similar research that intend to get as many practitioners as possible to appraise their perception in determining the most significant challenges or success factors when discussing certain phenomena. According to Kelley^79^ the data produced from survey can be generalized and are less expensive compared to other methods, as it reflect real-world observation.

The target population is Malaysian SMEs active in construction as listed in the official website^80^. Based on CIDB database a total population of 116,960 are considered as potential respondents. The random sampling technique is adopted to select the participants for this study. Determining the sample size was adopted from Enshassi and Al Swaity^81^ and Gouda Mohamed et al.^82^ as follows:

1 $$\:\mathrm{S}\mathrm{s}=\frac{\:{\mathrm{Z}}^{2}\:\mathrm{X}\:\mathrm{P}\left(1-\mathrm{P}\right)}{{\mathrm{C}}^{2}}$$

Where: Ss = sample size, Z = standardized variables (1.96 for 95% confidence level).

P = percentage picking a choice expressed as a decimal (used as 0.5), C= margin of error (8–9%),

$${\mathrm{Ss}} = \frac{{1.96^{2} \times 0.5\left( {1 - 0.5} \right)}}{{0.08^{2} }} = 118.6$$

$${\mathrm{New}}\;{\mathrm{ss}} = \frac{{{\mathrm{ss}}}}{{1 + \frac{{{\mathrm{ss}} - 1}}{{{\mathrm{pop}}}}}} = \frac{{118.57}}{{1 + \frac{{118.57 - 1}}{{116960}}}} = 119$$

The construction industry generally has a low response rate to surveys, with SMEs not an exception. The response rate in construction industry ranges from 20 to 30%^83^. To reach a number bigger than the minimum 119 responses, 590 questionnaires were distributed to potential respondents. In this study, among the 590 questionnaires sent to the selected sample, 268 of the returned questionnaires were valid representing approximately 45%of the response rate. This number of valid responses was sufficient to conduct statistical analyses, including factor analysis. Feedback then were transcript to IBM Statistical Package for the Social Sciences (SPSS) statistics software version 28.0 for the analysis^84^. The Mean Index (MI) value ranged from 1 to 5 is adopted in this research, and MI ≥ 3.5 is considered high^85^.The questionnaire survey was developed based on the challenges and enablers identified from based on expert perspective^30^. Before conducting the actual survey, a pilot study including forty-six Malaysian construction professionals were performed. The goal of conducting the pilot study is to ensure clearance, understanding, and relevancy of the research questionnaire content. According to the respondents’ input, the average time to finish a questionnaire was roughly 20 min. Throughout the pilot study, the researcher recorded participant opinions and perceptions of the survey’s vague words, usefulness and comprehension. Each respondent invited to express their level of agreement on the synthesized list of success factors and challenges using a five-point Likert-scale, where1 indicates “strongly disagree,” 2 “disagree,” 3 “neutral,” 4 “agree,” and 5 “strongly agree.” The questionnaire was distributed to around 590 participants, of which only 268 responses were deemed valid and suitable for further analysis, representing an excellent rate of 45%.

To evaluate the internal consistency of the gathered data, a reliability test employing Cronbach’s alpha (α) was carried out. The scale for internal consistency acceptance greater than 0.6, 0.7, and 0.9 is consider as acceptable, good, and excellent internal consistency^86^. In this research, the reliability test was performed on the data returned reported a Cronbach’s alpha (α) for the challenges factors is 0.936 and 0.984 for the success factors, which is considered excellent.

**Fig. 1:**
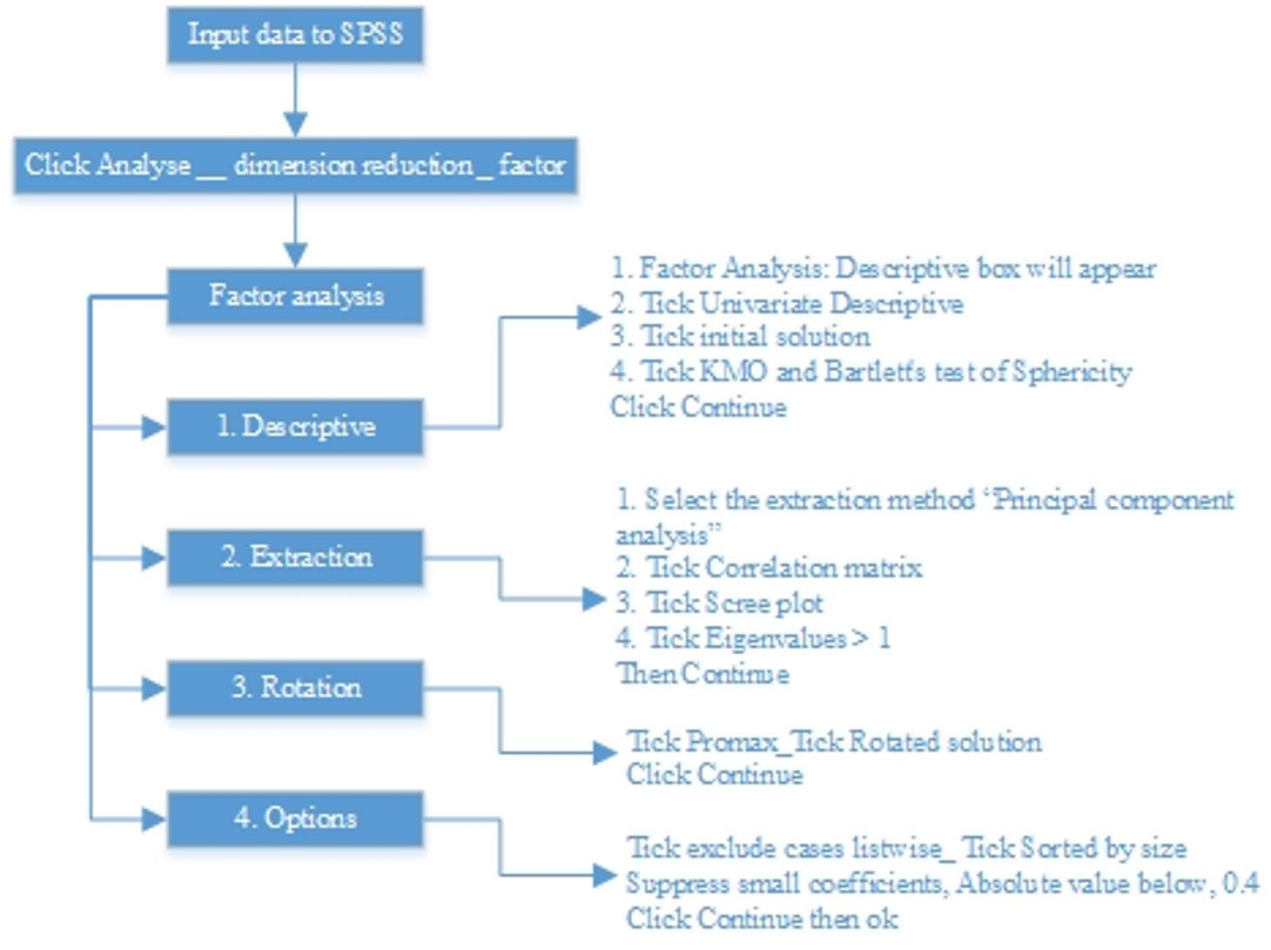
Procedures of factor analysis in SPSS.

Factor analysis in the SPSS statistical software was then utilized to reduce factors to a more manageable numbers and explore the underlying theoretical structure of the subject. This method, known as an Exploratory Factor Analysis (EFA), aims to represent interrelated factors with more general names^87^. In EFA, this study would identify the relationships and understand the structure within the factors. The EFA technique allows the software to suggest the optimum number of factors which can fit under one meaningful component^88^. The factors of BIM implementation scores are grouped based on Promax method of principal component analysis. This research adopted a suppress small coefficients with absolute value below 0.4 as reported in previous research^89,90^. This approach involves several steps in SPSS as described by Ferguson and Cox^91^ summarized in Fig. 1.

Exploratory Factor Analysis (EFA) offers two principal approaches for naming extracted factors: the post-analysis approach, commonly known as the recaptured item technique, and the pre-analysis approach, which utilizes marker variables and expert judgment to guide variable selection and naming^91^. This paper used pre-analysis approach technique, where the factors were firstly identified. Factor analysis using the principal component analysis (PCA) method is used to categorize the factors into three main components for the challenges and five main components for the success factors according to the analysis results. In order to determine a collection of variables that could tap those components, a group of experts were given definitions of the factors. Then the authors used an information from the literature to assist in judging and providing names of the new components. This study then adopted a focused meeting with 3 expert professionals from the industry to give a second judgement to the derived names of each component.

### Demographic analysis

The participants in this study were drawn from various construction companies in Malaysia, representing a diverse range of backgrounds within the industry. Table 3 presents the respondents’ demographic characteristics, classified according to their academic qualifications, business sector, experience, designations, and company establishment. Regarding educational qualifications, most of the professionals (46%) held a bachelor’s degree, followed by diploma and high school graduates, who constituted 24% and 14%, respectively. A smaller proportion of participants (10%) had attained a master’s degree, while only 1% possessed a Ph.D. In terms of business sectors, the respondents included professionals from a range of fields, including clients and developers, contractors, civil engineers, architects, mechanical, electrical, and plumping (MEP) engineers. Notably, contractors formed the largest group, comprising 45% of the respondents, followed by clients and developers at 20%. Engineering consultants accounted for 11% of the sample, while architects and other professionals, including academicians, represented 4% and 11%, respectively. This broad representation across various disciplines and organizational roles underscores the study’s comprehensive perspective on the construction industry. The diverse demographic composition enhances the validity of the findings by ensuring the inclusion of insights from key stakeholders within the Malaysian construction sector.

Table 3 further illustrates the respondents’ distribution according to the years of professional experience. Approximately 33% of the respondents reported having less than five years of experience, representing the largest group. Those with 5 to 10 years of experience accounted for around 27%, while respondents with over 15 years of experience made up 25% of the sample. The smallest proportion, 15%, consisted of respondents with 10 to 15 years of experience. Regarding job designations, the data indicates a diverse representation across different levels of management and professional roles. Executive management positions constituted 25% of the respondents, while 21% were from junior management, and 16% held senior management roles. The remaining 38% were classified as “others,” encompassing engineers, academicians, quantity surveyors (QS), and administrative personnel, making this the largest category. This broad range of roles suggests the inclusion of varied perspectives from individuals directly engaged in the construction industry.

**Table 3:** Demographic profile of respondents.

Demographic item	Category	Percentage (%)
Educational background	Doctorate	1%
	Master’s degree	10%
	Bachelor’s degree	45%
	Diploma	24%
	High-school	14%
	Others	5%
Business sector	Developer/Client	20%
	Engineering Consultant	11%
	Contractor	54%
	Architecture	4%
	others	11%
Work experience	< 5	33%
	5 to 10	27%
	10 to 15	15%
	> 15	25%
Designation	Executive	25%
	Senior Management	21%
	Junior Management	16%
	Others	38%
Company establishment	Public sector	32%
	Private sector	68%

The classification of respondents by the business sector further highlights the diversity of the participating organizations, reflecting the composition of the local construction sector. The designations spanned various domains related to civil or construction projects, including engineering, management, and academic roles within civil engineering departments. Notably, respondents were selected based on their knowledge of construction projects and Building Information Modeling (BIM), ensuring relevant expertise across the sample.

Concerning company establishment, participants were categorized as either from the public or private sector. The results show that 68% of respondents were from private sector companies, nearly double the 32% from the public sector. This is particularly significant given the Construction Industry Development Board (CIDB) of Malaysia’s mandate for the mandatory use of BIM in certain private-sector projects by 2020^92^. The strong representation from the private sector provides valuable insights into the industry’s responsiveness and readiness for BIM implementation in building projects across Malaysia. The reported results indicate that the majority of respondents possess adequate educational qualifications, and around 70% have experience more than five years, which strengthen the validity of this research findings.

## Results and analysis

### Awareness of BIM implementation

**Fig. 2:**
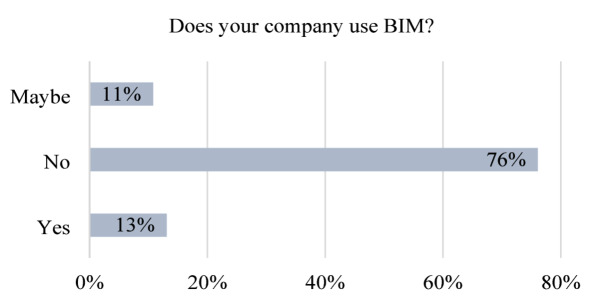
Level of BIM adoption among construction firms in Malaysia.

The findings presented in Fig. 2 indicate that only 13% of the surveyed participants reported using BIM technology within their organizations. Notably, most companies disclosed that BIM is not utilized across all building projects and is often adopted only when explicitly required. A substantial 76% of local construction organizations do not employ BIM applications, primarily due to the absence of BIM infrastructure and systems. This limited adoption can be attributed to several factors, including insufficient knowledge, the perceived high cost of implementation, lack of awareness, and the challenges associated with transitioning from conventional construction practices. These barriers are consistent with observations from previous reports, which have highlighted the persistently low levels of BIM adoption and implementation since 2013^33,93–95^. Additionally, approximately 11% of respondents were uncertain about whether their organizations were utilizing BIM, further underscoring the prevalent lack of knowledge and understanding regarding the technology.

**Fig. 3:**
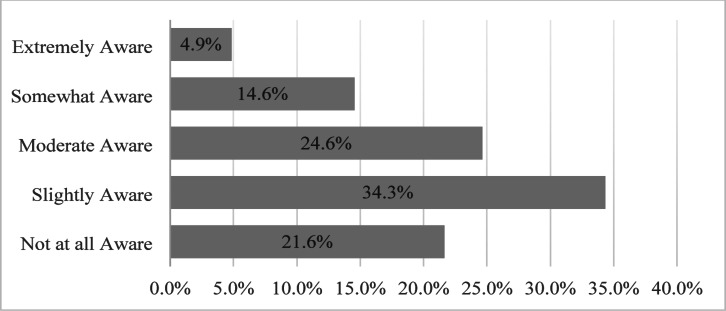
Awareness of BIM implementation process.

The level of awareness regarding BIM implementation processes significantly influences decision-making within top management, particularly when considering the adoption of BIM-based technological transformations. Implementing BIM in construction projects is inherently complex, requiring the integration of vast amounts of information from multiple stakeholders, particularly for small and medium-sized local enterprises. To assess participants’ awareness of the BIM implementation process, respondents were asked to evaluate their familiarity with BIM practices, as shown in Fig. 3. The results demonstrated that a considerable proportion of respondents possessed limited awareness. Specifically, 55.9% rated their awareness level below moderate, indicating a substantial knowledge gap. Furthermore, 24.6% of participants considered their awareness to be moderate, while only 19.5% regarded themselves as knowledgeable and well-informed about the BIM implementation process. Nevertheless, 21.6% were not aware at all. The feedbacks from the 21.6% respondents their feedbacks on the challenges were reported and. However, the feedback on the critical success factors were deemed irrelevant, the number of analyzed respondents were focused on the only BIM educated and experienced respondents. These findings emphasize the need for targeted awareness campaigns and capacity-building initiatives to foster greater understanding and adoption of BIM within the local construction sector.

The assessment of participants’ awareness levels in relation to BIM adoption within their organizations is summarized in Table 4. The results reveal that respondents from companies that have adopted BIM technology exhibit a significantly higher level of awareness regarding BIM implementation processes compared to their counterparts from non-adopting organizations. Notably, approximately 63% of professionals from BIM-adopting companies reported awareness levels above moderate. Conversely, among the respondents from organizations that have not adopted BIM, 60% rated their awareness below moderate, indicating they were only slightly aware or entirely unfamiliar with BIM processes.

To ensure data reliability, respondents were required to have direct involvement in construction projects and familiarity with BIM-related processes. Although respondents exhibited varying levels of professional experience, additional data screening procedures were implemented. Participants who entirely unaware of BIM were excluded from the analysis of critical success factors, ensuring that only informed perspectives contributed to the factor structure.

**Table 4:** Level of awareness to BIM application matrix.

Level of awareness to BIM application matrix	Application of BIM in organization			
	Yes 13%		No and maybe 87%	
	Frequency	%	Frequency	%
Not at all aware	2	6%	56	24%
Slightly aware	7	20%	*85*	36%
Moderate aware	9	26%	57	24%
Somewhat aware	9	26%	30	13%
Extremely aware	8	23%	5	2%
Total	35	100%	233	100%

Through data collection process, a recurring pattern was observed among respondents with lower levels of awareness. These individuals expressed uncertainty regarding BIM project execution, particularly concerning how project information would be effectively shared and coordinated across different organizations and stakeholders. Concerns were also raised about model ownership, data privacy, and the overall clarity of BIM execution procedures. To bridge this gap, several organizations have shown interest in enhancing their engagement with BIM by participating in relevant seminars and workshops. Additionally, numerous respondents emphasized the need for the government to provide a detailed and accessible guide outlining BIM execution plans. Such a resource would be particularly beneficial for small and medium-sized enterprises (SMEs), offering practical insights into the effective management of building projects using BIM processes. Furthermore, respondents suggested that the implementation of BIM should be mandated for specific project types across both public and private sectors to drive broader adoption and competency. The following points encapsulate the primary concerns and questions raised by respondents regarding BIM implementation:

- What: Concerns related to project information management and accessibility.
- Why: Questions about the intended use and benefits of BIM models.
- When: Uncertainty regarding project timelines and BIM deliverables.
- Who: Ambiguity surrounding the roles and responsibilities of stakeholders in BIM execution.
- Which: Uncertainty regarding the Level of Information (LoI) required at various project stages.
- How: Concerns about BIM processes, management, and execution methodologies.
- Where: Questions about data storage, management systems, and model accessibility.

Addressing these concerns through targeted educational initiatives, clear regulatory guidelines, and enhanced industry collaboration can significantly facilitate the wider adoption of BIM and improve its successful implementation within the construction sector.

### Challenges of BIM implementation

The integration of BIM technologies and advanced construction techniques has become a central focus for key stakeholders within the construction industry. Despite the competitive nature of the sector, the adoption of BIM remains relatively limited, particularly in mid- and low-rise building projects. This is especially evident among small and medium-sized enterprises (SMEs), which are responsible for the majority of such developments. These firms often encounter numerous challenges that hinder effective BIM implementation. A substantial portion of local construction companies have yet to adopt BIM systems, indicating that the industry is still in the early stages of fully embracing this technology. The key barriers to BIM adoption were identified through a systematic literature review and expert feedback, specifically within the context of building projects in the Malaysian construction industry.

A set of 20 challenges identified by Al-Ashmori et al.^30^ was adopted to assess their relevance and significance among small and medium-sized enterprises (SMEs) within the Malaysian construction industry. The full list of these factors is presented in Table 3. However, two challenges—CF14 (“Produce a BIM system guideline for technology implementation”) and CF19 (“Setting up an interoperability mechanism for notification and information sharing”)—were excluded from the survey investigation. Based on experts opinion referenced in Al-Ashmori et al.^30^, these challenges were deemed to be of lesser importance or not necessarily relevant within the study’s context.

Descriptive statistical analysis was conducted, with the challenges ranked according to their respective mean index, standard deviation, skewness, and kurtosis values, as presented in Table 5. This study utilized a valid sample size of 268 respondents. The data distribution was confirmed to be normal, with all skewness values falling within the acceptable range of ± 2, in accordance with the criteria outlined by Hair et al.^96^. The results indicate that “Development of execution procedures and legal frameworks for BIM implementation” (CF7) emerged as the most critical challenge, with a mean score of 4.08. “Standardizing BIM processes and defining implementation guidelines” (CF11) was identified as the second most significant challenge, recording a mean of 4.01 and a standard deviation of 0.74. “Creating affordable training programs” (CF8) ranked third, followed by “Building trust among BIM project teams” and “Bridging fragmented work processes and improving understanding of BIM technology and its implementation,” which ranked fourth and fifth, with mean values of 3.99 and 3.97, respectively. Additional challenges such as understanding BIM implementation, enhancing the decision-making process, fostering collaborative working environments, and coordinating BIM models were ranked seventh, eighth, and ninth, respectively.

**Table 5:** Summary of identified challenges: rankings, mean scores, standard deviations, skewness, and kurtosis values.

ID	Item	Rank	Mean	Std. Dev.	Skewness	Kurtosis
CF7	Development of execution procedure and legal frameworks for BIM implementation.	1	4.08	0.76	− 1.119	2.622
CF11	Standardizing BIM process and defining guidelines for its implementation.	2	4.01	0.74	− 1.122	2.794
CF8	Creating affordable training programs.	3	4.01	0.83	− 1.178	2.34
CF15	Building trust among BIM project teams and bridging the gap of work fragmentally.	4	3.99	0.74	− 0.929	2.298
CF10	Enhancing level of understanding of BIM technology and process implementation.	5	3.97	0.74	− 0.969	2.421
CF22	Boosting the decision-making process among stakeholders.	6	3.96	0.73	− 0.923	2.367
CF17	Understand BIM model interoperability mechanism among different BIM software.	7	3.96	0.75	− 1.008	2.64
CF12	Provision of comparative analysis between traditional and BIM-based projects as evidence.	8	3.93	0.81	− 0.891	1.386
CF20	Setting out an efficient mechanism for coordinating BIM models.	9	3.92	0.73	− 0.897	2.182
CF13	Overcoming the constraints of limited BIM software tools and compatibility issues.	10	3.92	0.78	− 0.903	1.664
CF21	Enhancing communication process among different parties.	11	3.91	0.7	− 0.966	2.716
CF9	Minimizing the initial costs associated with BIM implementation.	12	3.91	0.78	− 1.098	2.381
CF6	Build trust towards BIM technologies and overcome resistance factors.	13	3.9	0.76	− 0.794	1.609
CF16	Enhancing the Individual and group motivation to use BIM.	14	3.88	0.73	− 1.196	2.777
CF5	Convincing organizations and individuals to openly share information.	15	3.88	0.75	− 0.851	1.784
CF18	Creating a platform for a collaborative working environment.	16	3.86	0.69	− 0.917	2.683
CF3	Development of protocols for BIM standard modeling.	17	3.85	0.72	− 0.805	2.041
CF4	Developing a securing property assurance of BIM project information.	18	3.83	0.69	− 0.984	2.65
CF2	Utilization of current contracts to fulfill BIM projects requirements.	19	3.74	0.78	− 0.853	1.49
CF1	Creating demand for BIM projects or prioritizing BIM projects as a marketing brand.	20	3.7	0.78	− 0.815	1.267

The importance of these challenges indicates that BIM adoption difficulties among construction SMEs are not driven solely by technological limitations, but rather by weaknesses in processes, coordination, and organizational readiness. High-ranking challenges such as the lack of standardized BIM procedures, limited understanding of BIM processes, and fragmented collaboration reflect the informal and project-based nature of SME operations, where structured digital workflows are often absent. This suggests that BIM implementation within SMEs requires not only technological investment but also fundamental changes in project execution practices and inter-organizational coordination.

#### Factor analysis of challenges

Exploratory Factor Analysis (EFA) was conducted to identify the underlying dimensions among a set of 20 BIM-related challenges. This statistical technique is used to reduce a large number of observed variables into a smaller, more manageable set of latent factors, thereby revealing hidden structures and interrelationships within the data. The goal of the analysis is to maximize the shared variance and group correlated variables under common components.

Prior to conducting EFA, two preliminary tests were conducted to evaluate the adequacy of the data set for factor analysis: the Kaiser–Meyer–Olkin (KMO) measure of sampling adequacy and Bartlett’s test of sphericity. The KMO value was found to be 0.948, indicating excellent sampling adequacy. Additionally, Bartlett’s test was statistically significant (*p* = 0.001), confirming the presence of sufficient correlations among the variables to justify the application of factor analysis. These results support the suitability of the dataset, in alignment with guidelines suggested by Pallant^97^.

**Fig. 4:**
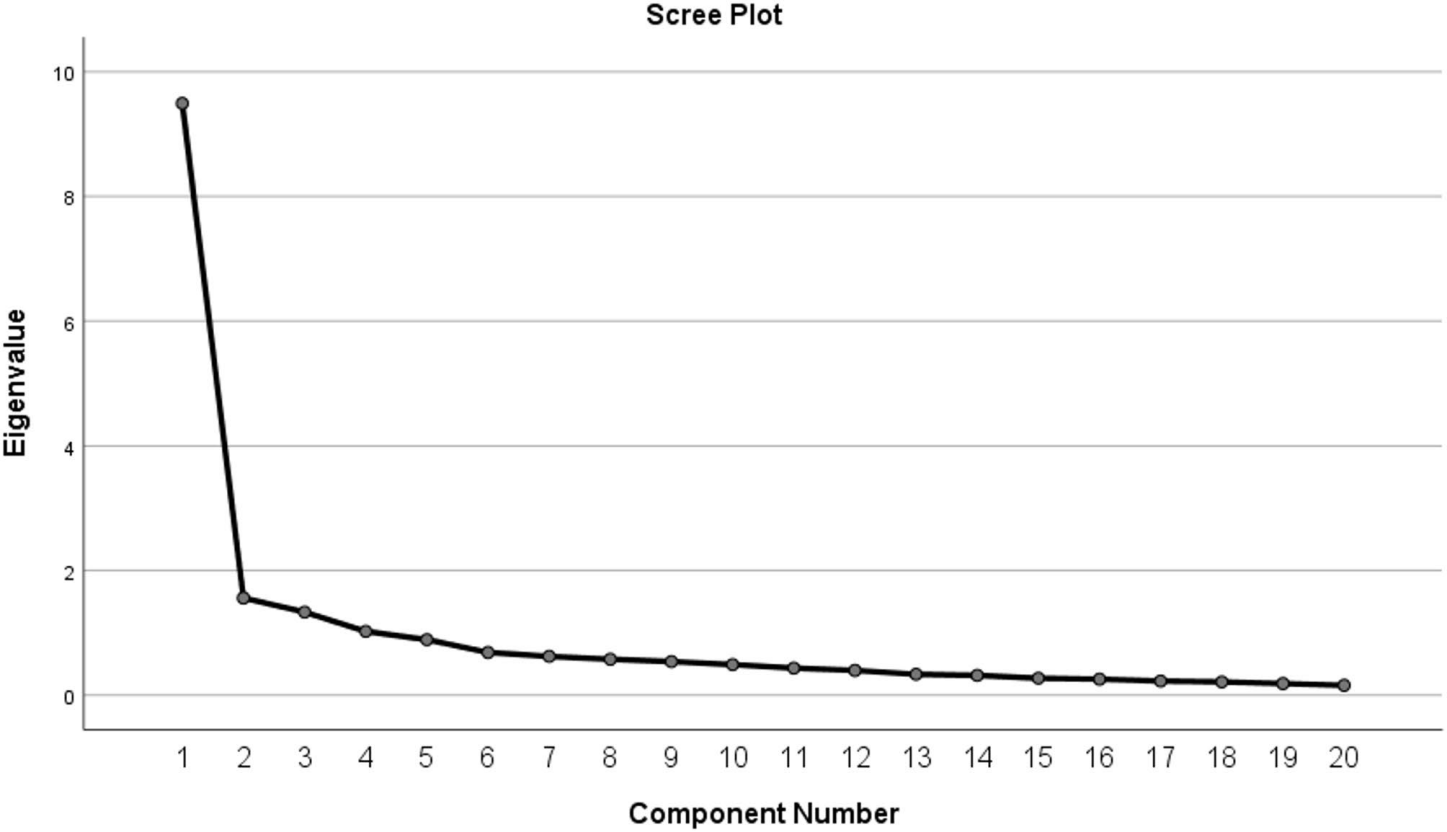
Scree plot diagram.

The factor extraction was performed using Principal Component Analysis (PCA). To improve the interpretability of the resulting factor structure, a Promax rotation—a type of oblique rotation—was applied, allowing for potential correlation among factors^97^. Based on the criterion of eigenvalues greater than 1, three distinct factors were extracted, with percent variance of 60.361, 5.680, and 4.884, respectively. These three components collectively accounted for 70.924% of the total variance, with the first factor alone explaining 60.361%. The breakdown of these three extracted components is presented in Fig. 4; Table 6.

**Table 6:** The identified challenges under exploratory factor analysis.

ID	BIM implementation challenge	Component			Rank	α	Name of component
		1	2	3			
CF11	Standardizing BIM process and defining guidelines for its implementation.	0.65			8	0.97	Processing
CF12	Provision of comparative analysis between traditional and BIM-based project as evidence.	0.53			10		
CF13	Overcoming the constraints of limited BIM software tools and compatibility issues.	0.56			9		
CF15	Build trust among BIM project teams and bridging the gap of work fragmentally.	0.78			6		
CF16	Enhancing the individual and group motivation to use BIM.	0.87			4		
CF17	Understand BIM model interoperability mechanism among different BIM software.	0.88			3		
CF18	Creating a platform for collaborative working environment.	0.85			5		
CF20	Setting out an efficient mechanism for coordinating BIM models.	0.78			7		
CF21	Enhancing communication process among different parties.	0.9			2		
CF22	Boosting the decision-making process among stakeholders.	0.95			1		
CF7	Development of execution procedure and legal frameworks for BIM implementation.		*0.88*		2	0.95	Organizational
CF8	Creating affordable training programs.		*0.99*		1		
CF9	Minimizing the initial costs associated with BIM implementation.		*0.69*		3		
CF10	Enhancing level of understanding of BIM technology and process implementation.		*0.53*		4		
CF1	Creating demand of BIM projects or prioritizing BIM projects as marketing brand.			0.72	4	0.93	Industrial
CF2	Utilization of current contracts to fulfil BIM projects requirements.			0.98	1		
CF3	Development of protocols for BIM standard modelling.			0.79	3		
CF4	Developing a securing property assurance of BIM project information.			0.84	2		
CF5	Convincing organizations and individuals to openly share information.			0.5	6		
CF6	Build a trust towards BIM technologies and overcoming resistance factors.			0.62	5		

The exploratory factor analysis grouped BIM challenges into three components—processing challenges, organizational challenges, and industrial challenges—demonstrating that BIM adoption barriers within SMEs are multi-dimensional. Processing challenges capture issues related to workflow integration, model coordination, and execution planning, indicating that SMEs struggle to align BIM processes with traditional construction practices. Organizational challenges reflect internal constraints such as limited expertise, resistance to change, and lack of managerial support, which directly affect SMEs’ readiness to implement BIM. Industrial challenges emphasize the influence of external factors, including the absence of regulatory enforcement, legal clarity, and industry-wide BIM standards.

The identification of these components provides a structured understanding of how BIM adoption difficulties manifest at different operational levels. Rather than existing in isolation, these challenges interact to compound implementation difficulties, suggesting that addressing BIM barriers within SMEs requires coordinated organizational, procedural, and industry-level interventions.

### Enablers (critical success factors) for effective BIM implementation in Building projects

The findings indicate a clear consensus on the importance of the enablers as essential elements for facilitating local small and medium enterprises (SMEs) in understanding BIM requirements and enhancing its implementation, particularly in mid- and low-rise building projects. The Content Validity Ratio (CVR) for all identified items exceeded the threshold value of 0.385, and the overall Content Validity Index (CVI) was calculated at 0.571, indicating acceptable validity. To ensure the reliability of the findings, responses were filtered based on participants’ self-reported awareness of BIM technology and processes. Respondents who indicated “not at all aware” were excluded from the factor analysis to ensure that the identified success factors reflected informed perspectives. Accordingly, out of 286 total responses, 210 valid responses were retained for the analysis of critical success factors.

Descriptive statistics for these enablers are presented in Table 7, with factors ranked based on their mean values. The most significant enablers identified were: “early involvement and participation of project teams” (SF17) and “the availability of information and technology” (SF29), both with a mean score of 4.05, followed closely by “early selection of appropriate BIM tools” (SF31) with a mean score of 4.04. Conversely, the least emphasized factors included the “creation of BIM-related business opportunities and market support” (SF5), and the “early formulation of collaborative methods among stakeholders” (SF42), each with a mean score of 3.92. The distribution of responses was assessed for normality, with skewness and kurtosis z-values recorded at -0.93 and 2.71, respectively, indicating an acceptable level of deviation from normality for further statistical analysis.

**Table 7:** Critical success factor mean, standard deviation, skewness, and kurtosis values.

ID	Item	Rank	Mean	Std. Dev.	Skewness	Kurtosis
CSF17	Early involvement and participation of project teams.	1	4.05	0.7	− 0.749	1.769
CSF29	Availability of information and technology.	2	4.05	0.68	− 1.151	3.812
CSF31	Early selection of the appropriate BIM tools to perform the task.	3	4.04	0.67	− 1.129	4.073
CSF18	Mutual trust, respect, and personal commitments to cooperation.	4	4.04	0.69	− 0.665	1.541
CSF25	Capability to use a BIM software tool.	5	4.04	0.72	− 1.201	3.668
CSF30	Early selection of adequate project delivery method.	6	4.03	0.69	− 0.997	3.09
CSF36	Produce models that can generate auto shop drawings for construction and fabrication.	7	4.03	0.71	− 1.007	2.834
CSF27	Ability to manage information in a structured manner in a 3D environment.	8	4.02	0.7	− 1.208	4.087
CSF11	Availability of BIM systems/ tools/ extensions to support BIM implementation.	9	4.02	0.64	− 0.671	2.264
CSF15	Collaboration and readiness to share knowledge, risks, and reward.	10	4.02	0.69	− 0.896	2.252
CSF21	Ability to define a suitable way to manage stakeholder needs and wants.	11	4.02	0.67	− 0.792	2.261
CSF22	Active communication systems with appropriate stakeholders.	12	4.02	0.67	− 0.792	2.261
CSF23	People’s knowledge and awareness of the BIM system and its application.	13	4.02	0.76	− 1.138	2.827
CSF26	Understanding the mechanism of BIM execution through the project life cycle.	14	4.02	0.72	− 1.044	3.27
CSF13	Insure continues development to fulfill technology participant expectations.	15	4.01	0.66	− 0.606	1.77
CSF16	Clear understanding of client requirements when using BIM in the project.	16	4.01	0.7	− 0.778	1.838
CSF34	Develop an intelligent 3D model that can be used by other disciplines.	17	4.01	0.74	− 1.089	2.622
CSF38	Produce accurate model-based documentation through the project lifecycle.	18	4	0.66	− 1.004	3.654
CSF35	Produce models with different levels of development LOD100-LOD500.	19	4	0.68	− 1.094	3.532
CSF1	Existence of procedures, frameworks, and guidelines.	20	4	0.69	− 1.15	3.574
CSF33	Design BIM coordination strategy among project parties.	21	4	0.69	− 1.226	4.269
CSF12	Availability of Securing intellectual property and cyber security of BIM outcomes.	22	4	0.7	− 0.598	1.347
CSF44	Identify and produce BIM deliverables at each phase of the project’s life cycle.	23	4	0.71	− 1.123	3.58
CSF9	Ability to accommodate changes and upgrade to BIM-based system.	24	4	0.75	− 1.192	3.222
CSF45	Determine and employ innovative ideas for collaborative practices.	25	3.99	0.7	− 0.986	2.858
CSF28	Knowing the usage of the multidisciplinary models that promotes collaborative processes.	26	3.99	0.71	− 1.11	3.521
CSF37	Visualize layout for site management, supervision, safety management, and quality management.	27	3.99	0.71	− 1.19	3.746
CSF39	To be able to identify risks associated with bidding BIM projects (types, size, teams, and locations).	28	3.99	0.74	− 0.966	2.214
CSF6	Readiness of government and organization to reward for self-development skill in BIM technology implementation.	29	3.99	0.77	− 0.745	1.34
CSF8	Top management support to implement BIM.	30	3.99	0.78	− 0.83	1.432
CSF43	Availability of effective project monitoring processes.	31	3.98	0.68	− 0.981	3.157
CSF32	Understanding BIM project scope and contract agreement.	32	3.98	0.71	− 1.213	3.908
CSF14	Knowledge and experience level of “players” in the BIM process and what are their drivers.	33	3.98	0.73	− 0.807	1.601
CSF19	Ability to define external stakeholders’ potential impact on projects.	34	3.97	0.69	− 0.994	2.954
CSF24	Ability to differentiate between different BIM software systems.	35	3.97	0.76	− 1.021	2.543
CSF4	Define team roles and responsibilities.	36	3.95	0.67	− 0.623	1.712
CSF3	Linking current policy with the BIM implementation requirement.	37	3.95	0.71	− 0.881	2.338
CSF10	Compatibility of BIM systems to support interoperability and collaboration.	38	3.94	0.71	− 0.9	2.471
CSF2	Develop research to identify changes with BIM implementation.	39	3.93	0.66	− 0.616	1.73
CSF20	Ability to understand each stakeholder’s interests.	40	3.93	0.68	− 0.832	2.09
CSF40	Availability of effective communication methods.	41	3.93	0.71	− 0.961	2.573
CSF41	BIM process re-engineering and decentralized decision-making.	42	3.93	0.71	− 1.261	3.72
CSF7	Ability to allocate sufficient financial resources to invest in BIM development.	43	3.93	0.8	− 0.906	1.731
CSF42	An early formulation for collaborative method between stakeholders.	44	3.92	0.71	− 1.092	3.29
CSF5	Create BIM business opportunities and market support.	45	3.92	0.76	− 0.801	1.601

The results further show that BIM implementation success within SMEs is strongly influenced by early project-stage decisions and collaborative practices. The SMEs benefit most when BIM requirements, roles, and responsibilities are clearly defined at the initial stages of a project. The importance assigned to professional skills and stakeholder interaction underscores the role of human capital and communication in achieving BIM implementation success. These findings suggest that BIM adoption within SMEs is not solely dependent on external mandates or technological availability, but on proactive planning, team coordination, and continuous skills development.

#### Factor analysis of BIM implementation enablers

This study employed Principal Component Analysis (PCA) to explore the underlying structure of enablers for BIM implementation. Factors were extracted based on the eigenvalue criterion, retaining only components with eigenvalues greater than 1, in accordance with the Kaiser rule. To improve the interpretability of the factor structure, the Promax oblique rotation method was applied. This approach supports the assumption that factors may be correlated and promotes a simple structure, whereby each factor is characterized by a small number of substantial loadings while minimizing cross-loadings^98,99^. A threshold loading value of 0.4 was used for item retention and interpretation. Although loadings of 0.3 are commonly accepted in the literature^98^, a higher threshold was selected in this study to reduce overlap and ensure greater clarity in factor distinctions^99^.

Prior to conducting factor analysis, data adequacy and suitability were verified through:

- The Kaiser–Meyer–Olkin (KMO) measure of sampling adequacy, which yielded a value of 0.943, indicating that the sample was highly suitable for factor analysis.
- Bartlett’s Test of Sphericity, which was statistically significant (*p* < 0.001), confirming that sufficient correlations existed among the variables to proceed with the analysis.

**Fig. 5:**
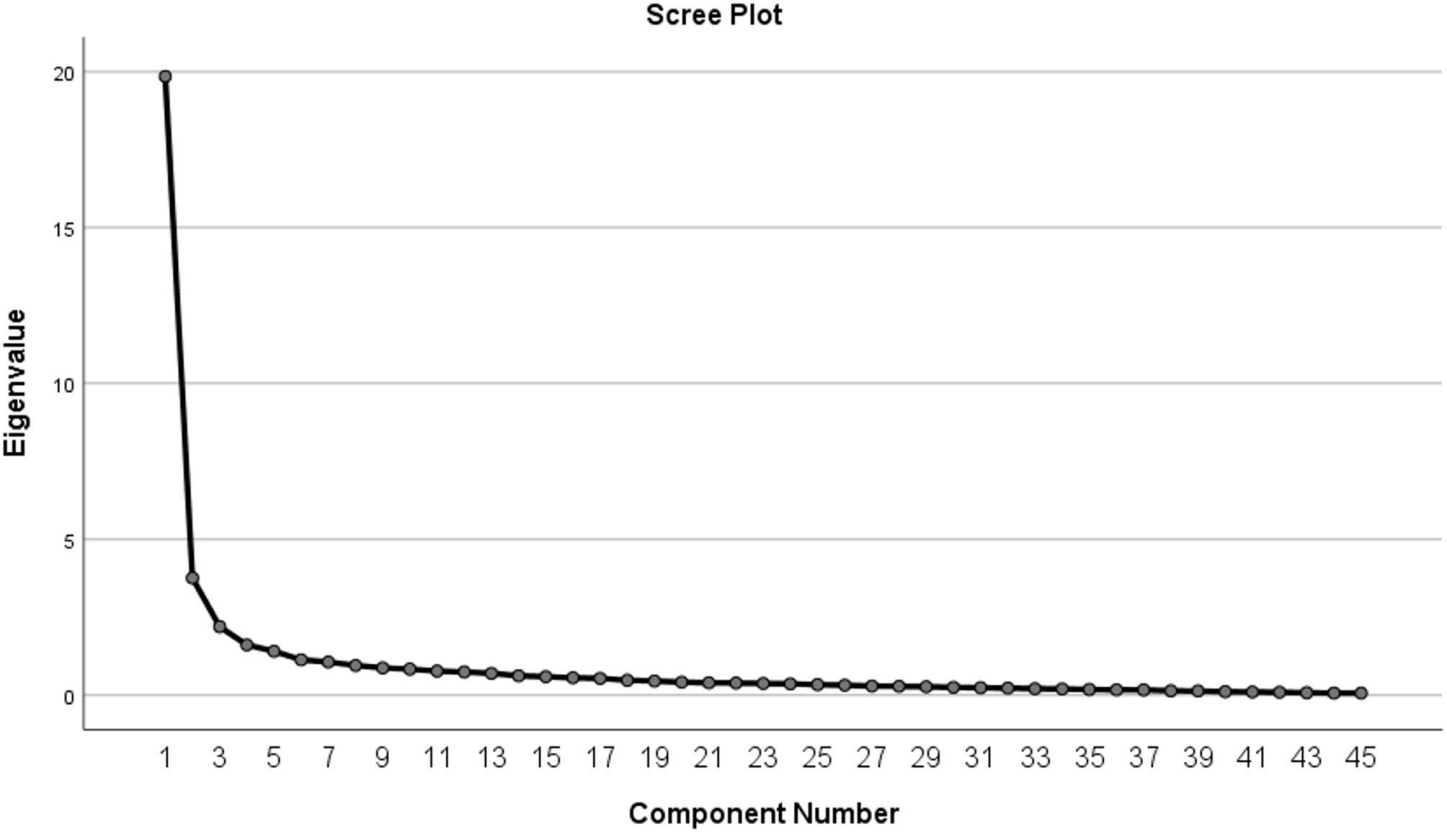
Scree plot diagram.

The communality values for all items ranged from 0.626 to 0.849, demonstrating acceptable levels of shared variance for inclusion in the factor model. Based on the PCA results, six clusters were initially extracted, collectively accounting for 74.77% of the total variance. After applying Promax rotation and utilizing the specified loading threshold of 0.4, five interpretable components were derived and are presented in Fig. 5; Table 8.

**Table 8:** Exploratory factor analysis for the identified success factors.

ID	Component					Rank	Cronbach alpha (α)	Name of components
	1	2	3	4	5			
CSF1	0.55					9	0.928	Policy Factors
CSF2	0.81					1		
CSF3	0.78					2		
CSF4	0.57					8		
CSF5	0.67					6		
CSF6	0.71					5		
CSF7	0.72					3		
CSF8	0.71					4		
CSF9	0.64					7		
CSF10		0.59				3	0.936	Technological Factors
CSF11		0.62				2		
CSF12		0.77				1		
CSF13		0.49				4		
CSF14			0.63			9	0.953	Stakeholders’ Interaction Factors
CSF15			0.88			2		
CSF16			0.77			4		
CSF17			0.69			6		
CSF18			0.69			7		
CSF19			0.81			3		
CSF20			0.88			1		
CSF21			0.74			5		
CSF22			0.65			8		
CSF23				0.42		8	0.951	Professionals’ Skill Factors
CSF24				0.40		9		
CSF25				0.63		6		
CSF26				0.68		4		
CSF27				0.62		7		
CSF28				0.65		5		
CSF29				0.88		1		
CSF30				0.77		3		
CSF31				0.80		2		
CSF32					0.65	11	0.967	BIM Processing Factors
CSF33					0.67	9		
CSF34					0.66	10		
CSF35					0.57	14		
CSF36					0.71	7		
CSF37					0.86	3		
CSF38					0.87	2		
CSF39					0.64	12		
CSF40					0.91	1		
CSF41					0.73	6		
CSF42					0.63	13		
CSF43					0.79	4		
CSF44					0.77	5		
CSF45					0.69	8		

The five extracted success-factor components—Policy Factors, Technological Factors, Stakeholder Interaction Factors, Professional Skill Factors, and BIM Processing Factors—provide an integrated view of the conditions required for effective BIM implementation in SME-driven projects. Policy and technological factors highlight the importance of supportive institutional environments and accessible digital infrastructure. Stakeholder interaction and professional skill factors emphasize collaboration and competency development, while BIM processing factors reinforce the need for structured execution planning and workflow management.

Collectively, these components demonstrate that successful BIM implementation within SMEs requires a balanced approach that integrates organizational readiness, human capability, and supportive external conditions. The structured grouping of these factors enhances understanding by translating individual success drivers into coherent implementation dimensions relevant to SME project environments.

## Discussion

Building Information Modeling (BIM) is increasingly recognized as a transformative solution to the limitations inherent in traditional construction^2^. The rapid pace of urbanization and infrastructure expansion has heightened the demand for residential developments. Simultaneously, there is a growing societal emphasis on the cultural, social, and environmental implications of buildings, particularly as the housing market matures and internal demand for integrated micro-environmental design solutions escalates^100^. As highlighted by Chong et al.^101^, the construction industry faces mounting expectations to deliver high-quality, sustainable, and efficient built environments. In pursuit of these objectives, the industry is progressively pivoting toward the adoption of BIM. This technology not only enhances the technical aspects of the construction process but also fosters a collaborative and interconnected work environment, ultimately improving project productivity and sustainability across the entire life cycle^11^. Consequently, BIM implementation has become an essential consideration in the industry’s strategic development agenda.

Nonetheless, BIM implementation is a complex endeavor, requiring ongoing identification and evaluation of success factors to address its multifaceted challenges. Al-Ashmori et al.^30^ emphasize that BIM is seldom employed in mid- and low-rise building projects, especially within developing nations. Typically, only large and mega construction firms consistently adopt BIM, often in response to client-specific demands. Although previous studies have identified challenges and critical success factors based on expert perspectives^30^, there remains a need for empirical validation across varying firm sizes. To address this gap, the present study offers a comprehensive analysis of BIM implementation enablers, with a particular focus on Small and Medium Enterprises (SMEs) in Malaysia.

The findings reveal that the enablers of BIM implementation can be categorized into five distinct components, derived from Exploratory Factor Analysis (EFA), as follows:

- Component 1 – Policy Factors: This category encompasses institutional and regulatory aspects, including contractual frameworks, strategic business opportunities, executive leadership support, accessibility to financial resources, organizational flexibility to adapt to changes, and the readiness of governmental and institutional bodies to build BIM-related competencies. Consistent with Olanrewaju et al.^17^, this paper find that the presence of policy enforcement (such as mandatory BIM use in public projects) significantly correlates with adoption success. The SMEs in this study view government incentives and regulatory clarity as essential to reducing uncertainty and cost risk, aligning with international experiences in Singapore and Hong Kong where policy mandates accelerated adoption. Although Malaysia has adopted a policy mandating the use of BIM, it applies only to large construction projects, thereby excluding SMEs to benefit of such policies. These elements collectively reflect the policy-level infrastructure required for effective BIM integration for SMEs.
- Component 2 – Technological Factors: This component relates to the technical readiness and infrastructure supporting BIM deployment. It includes tool compatibility, interoperability, ease of access for end-users, data security considerations, and technological alignment with user needs. These factors underscore the foundational role of a robust digital ecosystem in facilitating BIM implementation. The SMEs perceive technology not as an innovation barrier per se, but as a cost and interoperability challenge. Similar patterns are seen in Abubakar et al.^10^, where limited access to compatible platforms constrained BIM utilization. Thus, low-cost, open BIM, and cloud-based solutions could enable SMEs to bypass high software and infrastructure barriers.
- Component 3 – Stakeholder Interaction Factors: This component captures the relational and collaborative dynamics among project stakeholders. It includes stakeholders’ knowledge and experience with the BIM process, their willingness to collaborate, share risks and rewards, mutual trust and respect, awareness of others’ influence on project outcomes, and alignment of individual interests. These elements highlight the importance of effective communication, coordination, and role clarity among all project participants. This is directly linked to the Processing Challenges cluster, interpreting it as the relational antidote to process fragmentation. This insight is supported by Zahrizan et al.^33^, who stressed early coordination among project actors as pivotal for BIM success. The SMEs, which often work in less formalized project networks, rely more heavily on interpersonal trust and communication quality to enable BIM collaboration.
- Component 4 – Professional Skill Factors: This component focuses on individual competencies related to BIM tools and practices. It encompasses professionals’ awareness, technical proficiency, familiarity with BIM execution strategies, and their capacity to collaborate in a 3D modeling environment throughout the project life cycle. These factors emphasize the necessity of skill development and training for effective BIM usage. This factor is the capacity dimension of BIM adoption, bridging both organizational and industrial barriers. Previous studies (e.g., Aranda-Mena et al. and Chan^29,36^ consistently identify skill deficits as a key limitation, but this paper findings show that training availability is more decisive than technological readiness for SMEs. This provides new empirical evidence that human capital investment yields greater adoption leverage in SME environments.
- Component 5 – BIM Processing Factors: Comprising 14 key items, this component covers process-related aspects of BIM implementation. It includes defining the project scope, formulating coordination strategies, establishing model deliverables and levels of detail, employing visualization techniques, managing project risks, planning communication, and monitoring execution. These elements reflect the procedural framework needed to integrate BIM throughout a project’s life cycle. This component is the operational backbone of successful BIM deployment, directly countering Processing Challenges. The discussion compares our findings with Succar^75^, emphasizing that structured workflows and shared modeling standards enhance efficiency. Specifically, the SMEs require simplified but standardized BEPs tailored to small-scale projects, differing from complex frameworks used by large firms.

In addition to these enablers, the study identified three primary categories of challenges that hinder effective BIM implementation. The components are referred to as Processing challenges (component 1), Organizational challenges (component 2), and Industrial challenges (component 3) based on the thematic analysis of the variables. These were derived through EFA and collectively explain the empirical structure of 20 core challenges:

- Component 1 – Processing Challenges: Accounting for 60.361% of the total variance, this component reflects significant operational challenges in BIM workflows. These include uncertainties regarding BIM execution timing, coordination gaps, stakeholder mistrust, and inadequate decision-making frameworks. These items represent operational and coordination deficiencies that directly affect BIM workflow efficiency. Zahrizan et al.^33^ similarly reported that Malaysian firms often lack standardized BIM execution procedures and communication protocols. However, our study extends this understanding by revealing that these process-related barriers are more acute in SMEs, which typically lack in-house BIM coordinators or standardized BEPs (BIM Execution Plans). That mean SMEs experience BIM as a procedural challenge rather than purely a technological one—indicating that improving interoperability and communication frameworks could have the highest practical impact for this group.
- Component 2 – Organizational Challenges: The second component clusters items such as lack of affordable training, insufficient standardization, and unclear distinctions between traditional and BIM-enabled workflows. These factors represent crucial impediments to institutionalizing BIM within organizations. These represent internal readiness constraints; human resource and management limitations that prevent SMEs from integrating BIM into daily practice. Abubakar et al.^10^ similarly reported in Nigeria that small firms lack adequate BIM knowledge and financial flexibility. The revised text notes this cross-country consistency, reinforcing the argument that these issues typify SME barriers in developing economies.
- Component 3 – Industrial Challenges: The third component addresses broader industry-level constraints, including weak demand for BIM, outdated contractual frameworks, absence of standardized BIM modeling protocols, concerns about data security, and insufficient trust in model sharing among industry stakeholders. Addressing these systemic challenges is vital for widespread BIM adoption across the sector.
- These macro-environmental limitations that suppress BIM diffusion across the SME ecosystem. This component parallels findings from Ghaffarianhoseini et al.^49^, who emphasized the absence of supportive policy frameworks and weak client demand as deterrents to BIM uptake in developing regions. Our study, however, contributes new insight by showing that SMEs perceive these external factors as beyond their control, which underscores the need for policy-driven and client-oriented incentives to stimulate BIM adoption at scale.

The identification of five success-factor components and three challenge components is consistent with previous BIM frameworks, yet extend it to another perspective. The current findings provide a tailored structure to SME operational realities, whereas previous studies classified challenges broadly as technological, organizational, or policy-related. This demonstrates that BIM implementation in SME is driven less by software capability and more by process integration, skills development, and stakeholder coordination.

## Conclusion

This research was established to empirically investigate the challenges and critical success factors (CSFs) influencing BIM implementation within SMEs in Malaysia. Upon the factors evaluated by Al-Ashmori et al.^30^, this research designed in a questionnaire and distributed it randomly to 590 local organizations, yielding 268 valid responses. The study applied demographic profiling, descriptive statistics, and reliability analysis, all of which confirmed the data’s adequacy and enabled systematic categorization of BIM-related challenges and success factors. The finding indicates that BIM adoption among Malaysian SMEs remains very limited. Only 13% of respondents reported active in BIM usage, while 76% reported no current BIM use, and 11% of respondents were unsure if their companies were using BIM. This highlights the widespread lack of knowledge and understanding of the technology. These findings reaffirm that BIM adoption among SMEs remains a pressing national and industry challenge that requires an immediate intervention.

The study highlights that implementing BIM in building projects continues to pose considerable challenges for industry stakeholders. In particular, the identified challenges and success factors are directly useable for SMEs working in mid- and low-rise building projects which remains limited. Key challenges include the need for affordable training programs, minimizing initial costs, understanding BIM technology and process, creating a collaborative working environment, coordinating BIM models, and standardizing BIM process. These challenges were ranked according to their importance and analyzed using factor analysis, which revealed three main factors: processing challenges, organizational challenges, and industrial challenges. Overcoming these challenges is critical for advancing BIM implementation across the sector.

On the other hand, the study also identified the critical success factors that facilitate effective BIM implementation, particularly among participants with sufficient awareness of BIM technology and process. The most critical success factors include: early engagement of project stakeholders, availability of relevant information and technologies, and early selection of appropriate BIM tools. These factors were organized into five principal categories: Policy Factors, Technological Factors, Stakeholder Interaction Factors, Professional Skill Factors, and BIM Processing Factors. The identified factors are directly useable for SMEs and policy makers, whereby addressing and enhancing these areas will be essential for successful BIM integration into low and mid-rise construction projects in Malaysia.

Overall, this study contributes to BIM implementation literature by offering empirically validated insights from the perspective of construction SMEs in a developing country context. Unlike previous studies that focus on large firms or conceptual discussions, this research identifies and statistically validates three BIM challenge components and five success-factor components specific to SME-driven building projects. The findings extend existing BIM frameworks by incorporating organizational scale and resource constraints, offering a structured foundation for future SME-focused BIM research and policy development.

### Conceptual and empirical contributions

This study contributes both conceptually and empirically to the understanding of BIM implementation in developing countries, particularly SMEs within the construction sector. By identifying and empirically validating the critical challenges and success factors associated with BIM adoption, the research offers a foundational framework upon which future studies can build. The findings provide valuable insights for policymakers and government entities, enabling them to formulate strategic action plans that address the barriers when implementing BIM in construction industry.

Furthermore, this study advances theoretical development by offering a mathematically grounded model that highlights the key challenges impeding BIM adoption. These constructs can be leveraged to prioritize intervention areas and develop targeted policies. While a sufficient literature exists on BIM implementation in developed countries, research targeting SMEs companies in developing contexts such as Malaysia remains limited. This study addresses this gap by offering empirical evidence and theoretical insight, thereby enriching the global discourse on BIM implementation in underrepresented field.

### Managerial implications

Although the successful implementation of BIM is often perceived as complex and resource-intensive, this study provides practical implications that are directly relevant to construction managers, clients, contractors, and consultants. The empirical results offer actionable insights into the primary obstacles and enablers of BIM adoption in building projects, particularly those typical in the Malaysian construction industry, paving the way for effective BIM implementation.

Key managerial takeaways include:

- Proactive Challenge Mitigation: Identifying and addressing critical BIM implementation challenges enables AECO firms to mitigate risks early in the project lifecycle, ultimately enhancing project efficiency and client satisfaction through better design visualization and coordination.
- Informed Decision-Making: The analysis of BIM-related challenges and CSFs supports evidence-based decision-making, offering construction stakeholders a clearer understanding of their organizational readiness and areas needing improvement.
- Strategic Development: The findings serve as a guide for developing organizational strategies that align with technological advancements and sustainability goals, thereby fostering a more competitive and adaptive construction environment.

Additionally, the study lays the groundwork for future research that can apply structural modeling techniques, such as path analysis, to explore causal relationships among the identified CSFs and further develop implementation framework.

### Limitations and future research

While this study offers meaningful insights into the implementation of BIM, it is important to acknowledge several limitations that may influence the scope and applicability of its findings. Although the empirical data of this study are Malaysia-specific, the challenges and success factors is theoretically universal and applicable to SME-dominated construction sectors in other developing countries. The full generalizability to all global contexts, particularly developed economies, should not be assumed. However, the study lays a foundation for comparative replication. Future cross-country studies using the same instrument across multiple developing economies are needed to empirically test the universality of the proposed components. Moreover, further studies should consider expanding the sample to include multiple regions or countries, thereby enhancing the external validity of the results. Second, the research is conducted in a cross-sectional manner, lacking the exploration of organizational and historical contexts related to BIM implementation. Longitudinal research is recommended to explore how BIM adoption challenges and success factors interact over time and influence project outcomes.

By addressing these limitations, future research can build a more comprehensive and robust understanding of BIM implementation. Such advancements will not only benefit academic scholarship but also provide valuable guidance for practitioners aiming to improve BIM integration in construction processes.

## *Supplementary Information*

Below is the link to the electronic supplementary material.

Supplementary Material 1

Supplementary Material 2

## Data Availability

The datasets used and/or analyzed during the current study are available from the corresponding author on reasonable request.
